# A dual-functional sulfone biscompound containing 1,2,3-triazole moiety for decolorization and disinfection of contaminated water

**DOI:** 10.1007/s11356-022-20932-5

**Published:** 2022-06-08

**Authors:** Emad K. Radwan, Huda R. M. Rashdan, Bahaa A. Hemdan, Asmaa A. Koryam, Mehrez E. El-Naggar

**Affiliations:** 1 Water Pollution Research Department, National Research Centre, 33 El Buhouth St, Dokki, Giza, 12622 Egypt; 2 Chemistry of Natural and Microbial Products Department, Pharmaceutical and Drug Industries Research Institute, National Research Centre, 33 El Buhouth St, Dokki, Giza, 12622 Egypt; 3 Institute of Textile Research and Technology, National Research Centre, 33 El Bohouth St, Dokki, Giza, 12622 Egypt

**Keywords:** Adsorption, Cationic dyes, Kinetic and isotherm models, Multi-functional materials, Antimicrobial activity

## Abstract

Water decontamination from toxic dyes and pathogenic microorganisms is critical for life on Earth. Herein, we report the synthesis of sulfone biscompound containing 1,2,3-triazole moiety and evaluation of its dye decolorization and biocidal and disinfection efficiencies. The decolorization efficiency was tested under different experimental conditions, while the biocidal action was examined against various types of waterborne pathogens, and the disinfection of some pathogenic microbes was executed in artificially contaminated water. The findindgs illustrated that the solution initial pH (pH_*i*_) affected the decolorization efficiency significantly. About complete removal of 10 mg/L malachite green (MG) dye was achieved after 10 min using 3 g/L of the sulfone biscompound at pH_*i*_ 6. The pseudo-second-order equation suited the adsorption kinetics accurately, while the equilibrium data was suited by Langmuir isotherm model. Electrostatic, n–π, and π–π interactions brought about the adsorption of MG onto the sulfone biscompound. The biocidal results indicated that the sulfone biscompound had a powerful antibacterial potential against the tested bacterial species. Likewise, the distinction trail revealed that after 70–90 min of direct contact with an effective dose, the tested pathogens could be completely eliminated (6-log reduction). Overall, the newly synthesized sulfone biscompound can efficiently remove cationic dyes and disinfect contaminated water.

**Supplementary Information:**

The online version contains supplementary material available at 10.1007/s11356-022-20932-5.

## Introduction

Water is one of the basic constituents of life. Recently, the amount of wastewater has increased due to the population growth, industrial development, and increased agricultural and urbanization activities. The discharge of wastewater to the aquatic environment introduces various chemicals such as dyes and pigments, metals, organics, pharmaceuticals, and pathogenic microorganisms which deteriorate the water quality and threaten the life on Earth (Alene et al. 2021; Igwegbe et al. 2021; Rubio-Clemente et al. 2021; Tohidi et al. 2021). Among these pollutants, synthetic dyes attracted the researchers’ interest because of their application in various industries, high visibility, resistance to biodegradation, reduction of the photosynthesis process, toxicity, mutagenicity, carcinogenicity, and allergenic effect (Alene et al. 2021; Güler et al. 2021; Rubio-Clemente et al. 2021; Sartape et al. 2017; Shojaei et al. 2021; Tohidi et al. 2021). Malachite green (MG) is one of the synthetic dyes that is widely used in aquaculture, textile, leather, paper, and food industries (Alene et al. 2021; Bekçi et al. 2008; Lin et al. 2021; Rubio-Clemente et al. 2021; Shojaei et al. 2021; Tohidi et al. 2021). It is a low-cost and easily available basic (cationic) N-methylated diamino triphenyl methane dye (Figure S1).

MG poses several serious risks to the environment and public health (Alene et al. 2021; Bekçi et al. 2008; Güler et al. 2021; Rubio-Clemente et al. 2021; Sartape et al. 2017; Shojaei et al. 2021). For example, it is biologically stable and acutely toxic to a bunch of aquatic and terrestrial creatures. Also, it is reported to be a multi-organ toxin, carcinogenic, mutagenic, teratogenic, affects the immune and reproductive systems, and enhances the liver tumor. In response to these effects, numerous countries have banned MG and the US Food and Drug Administration is not approving it (Bekçi et al. 2008; Sartape et al. 2017). However, MG still used in several parts of the world (Lin et al. 2021; Rubio-Clemente et al. 2021; Sartape et al. 2017) and traditional treatment processes are ineffective in its removal (Güler et al. 2021; Rubio-Clemente et al. 2021; Sartape et al. 2017). Therefore, developing efficient treatment process to remove MG and dyes, generally, is still a challenging issue worldwide. Numerous approaches have been explored to remove dyes from water (Abdel-Karim et al. 2021; El Malah et al. 2021; Margha et al. 2020). By far, adsorption is the most commonly applied technique for the removal (decolorization) of dyes from water (Alene et al. 2021; Bekçi et al. 2008; Güler et al. 2021; Nour et al. 2021; Shojaei et al. 2021). The decolorization of dye-contaminated water by adsorption technique ensures the removal of the dyes without the formation of toxic by-proudcts that could be formed by the degradation techniques. The structural features of the adsorbent define the effectiveness of the adsorption process (Abdel Ghafar et al. 2020; Tohidi et al. 2021).

On the other hand, the density of microbial communities in receiving water streams increases by wastewater discharge. Waterborne pathogens and non-fatal infections are accountable for nearly two hundred million decease each year (Ahmad et al. 2019). Some opportunistic microbes such as *Pseudomonas aeruginosa*, *Legionella pneumophila*, and *Mycobacterium avium* are responsible for an emerging waterborne infection issue with a severe yearly financial charge (Falkinham et al. 2015). Chlorination is the most superior disinfection technique since chlorine has forceful antimicrobial action versus various disease-causing bacteria (Reddy and Elias, 2021). However, chlorination has certain shortcomings. The formation of cancer-causing substances disinfection by-products (DBPs) such as trihalomethanes and haloacetic acids has been the most problematic (El Nahrawy et al. 2019). Furthermore, the presence and increase of some chlorine-resistant organisms in water is another shortcoming (Luo et al. 2021). As a result, a new disinfection approach that does not produce DBPs and is more effective against chlorine-resistant species is highly demanded (Abou Hammad et al. 2020).

Fused nitrogenous heterocyclic analogues especially 1,2,3-triazoles exhibit extraordinary performance in variuos fields ranging from biomedical applications (El-Naggar et al. 2020; Rashdan 2019; Rashdan et al. 2021b, a), catalysis (Thomas et al. 2016; Zheng et al. 2015), supramolecular chemistry (Schulze and Schubert, 2014), flourecent imaging (Pedersen and Abell, 2011), polymer chemistry, and material science applications (Agouram et al. 2021, Hiba and Sreekumar, 2021, Phukan et al. 2021, Sonawane and Pore, 2022, Wang et al. 2021). Owing to their excellent properties like aromatic character, easy synthesis, exceptionally excellent yield of the end product, high chemical stability, hydrogen bonding ability, and strong dipole moment (4.8–5.6 Debye) (da SM Forezi et al. 2021, Jiang et al. 2021), 1,2,3-triazoles have gained a great deal of attention in industry and academics. Recently, 1,2,3-triazole-rich molecules revealed much importance in the field of water and wastewater management owing to their strong antimicrobial properties along with antifouling nature of the triazole ring and their potential application in functionalization of versatile inorganic moieties like carbon nanoparticles, metal oxide nanoparticles, etc. (Kuznetsov 2021).

The sulfone biscompound may offer efficient water and wastewater management approaches due to their triazole rings and sulfone groups. These functional groups endow the compound a substantial biocidal action against a broad range of pathogenic microbes, relying on the production of reactive oxygen species (ROS) and the destruction of cell wall integrity upon straight interaction (Aljerf et al. 2021). Thus, such compounds offer a powerful and worthy substituted approach for conventional disinfectants (Kokkinos et al. 2020). Also, the functional groups of the sulfone biscompounds could act as potential adsorption sites for chemical contaminants.

The aim of this work is to prepare a new sulfone biscompound containing 1,2,3-triazole moiety and evaluating its performance for the removal (decolorization) of MG as a model for cationic dyes from aqueous medium at different conditions such as solution initial pH and sulfone biscompound dosage. The adsorption kinetic and isotherm were tested and modeled using different models as well. Furthermore, some waterborne pathogens were disinfected using the prepared sulfone biscompound.

## Materials and methods

### Chemicals

The 4,4′-sulfonylbis(azidobenzene) (SBAB), acetyl acetone, methanol, and sodium methoxide were purchased from Sigma-Aldrich Co. (USA) and used as received without further purification.

### Synthesis of 1,1′-((Sulfonylbis(4,1-phenylene)) bis(5-methyl-1H-1,2,3-triazole-1,4-diyl))bis(ethan-1-one) (SBPTE)

Two milliliters (20 mmol) of acetyl acetone and 3 g (10 mmol) of SBAB were dissolved in 20 mL methanol containing 1 g of sodium methoxide; then, the mixture was stirred under reflux for 5 h. After cooling, the resulting solid was collected and recrystallized from ethanol to get a white powder, m.p. 248–250 °C; yield (95%); FT-IR (KBr, cm^−1^): *v* 2919, 2852 (CH), 1617 (C = C); H^1^NMR (500 MHz, DMSO-d*6*): *δ* 2.36 (s, 6H, 2CH_3_), 2.42 (s, 6H, 2CH_3_), 7.75 (d, 4H, *J* = 10.0 Hz, ArH), 8.11 (d, 4H, *J* = 10.0 Hz, ArH); C^13^NMR (100 MHz, DMSO-d*6*): *δ* 9.80, 27.69, 126.65, 129.43, 138.1f9, 139.21, 141.50, 143.07, 93.29.

### Characterization of the materials

The melting point of the synthesized compound was determined using an electrothermal apparatus and was not corrected. H^1^NMR and C^13^NMR spectra were recorded in (CD_3_)_2_SO solutions on a BRUKER 500 FT-NMR system spectrometer. The chemical shifts were measured in ppm (*δ*) related to TMS (0.00 ppm).

The powder of the synthesized SBPTE before and after MG dye adsorption was directly deposited on double-sided carbon tape then gold coated. Scanning electron microscopy (SEM) was examined using a scanning electron microscope (FE-SEM QUANTA FEG250, Republic of Czech). The SEM was operated with a secondary detector at 30 kV. After importing SEM images into the Gwyddion 2.4 programme, 3D roughness images and roughness parameters were generated.

### Assessment of the adsorption properties

The adsorption characteristics of SBPTE were evaluated using batch adsorption method using MG dye as a model for cationic dyes. Figure S1 shows the chemical structure and some features of MG dye. To determine the optimum initial pH (pH_*i*_) of the solution, 100 mg of SBPTE was suspended in 100 mL of MG solution (10 mg/L) preadjusted to pH_*i*_ 2, 4, 6, and 8 and agitated at 300 rpm and ambient temperature. Samples were collected after 10, 20, 30, 45, and 60 min, and the residual concentration of MG was determined using JASCO V730 (Japan) UV–Vis spectrophotometer at a maximum wavelength of 617 nm. The percentage removal (*R*%) of MG was calculated by Eq. 1.

1 $$\mathrm{R}\%\,=\,\left(1\,-\,\frac{C_t}{C_i}\right)\,=\,100$$

where *C*_*t*_ and *C*_*i*_ are the concentrations of MG dye after time *t* and zero, respectively.

The effect of SBPTE amount on the adsorption process was explored by shaking 100, 200, 300, and 400 mg of SBPTE in 100 mL of MG solution (10 mg/L) preadjusted to pH_*i*_ 6. Samples were withdrawn at predetermined period, and the amount of MG adsorbed per unit mass (*q*_*t*_, mg/g) of SBPTE was calculated by Eq. 2.

2 $${q}_{t}{ = }\frac{\left({C} {o} - {C}{t}\right) {V}}{m}$$

where *V* (L) and *m* (g) are the volume of MG dye solution and weight of SBPTE, respectively.

The effect of *C*_*o*_ of MG and adsorption isotherm were investigated by contacting 300 mg of SBPTE with 100 mL MG solution of *C*_*o*_ 5, 10, 15, 20, and 40 mg/L for 1 h. The residual concentration of MG was determined and *R*% and *q*_*e*_ were calculated.

#### Adsorption kinetics modeling

The kinetics data was examined using the non-linear forms of the pseudo-first-order (PFO; Eq. S1) (Langergren and Svenska, 1898) and pseudo-second-order (PSO; Eq. S2) (Blanchard et al. 1984) models.

#### Adsorption isotherm modeling

The adsorption equilibrium data were analyzed by the non-linear forms of Freundlich (Eq. S3) (Freundlich 1906), Langmuir (Eq. S4) (Langmuir 1918), Dubinin–Radushkevich (D–R; Eq. S5) (Dubinin and Radushkevich, 1947), Temkin (Eq. S6) (Temkin and Pyzhev, 1940), and Redlich–Peterson (R–P; Eq. S7) (Redlich and Peterson, 1959) models.

#### Adsorption models fitting

OriginPro 2016 software version 9.3.226 with user-defined fitting functions was utilized to perform the non-linear fit of the investigate kinetics and isotherm models to the experimental data. It also was used to calculate the parameters of the models and their corresponding error functions. The chi-square (*χ*^2^) was minimized using damped least-squares method. The goodness of fitting was judged based on the values of coefficient of determination (*R*^2^; Eq. S8), *χ*^2^ (Eq. S9), and the root-mean-square error (RMSE; Eq. S10). High value of *R*^2^ and low values of *χ*^2^ and RMSE indicate that the model can reliably and accurately describe the experimental data.

### Assessment of the antibacterial activity

Six different types of waterborne pathogenic bacteria, specifically four Gram-negative (*E. coli* O157:H7 ATCC 35,150, *Salmonella enterica* serovar Typhimurium ATCC 14,028, and *Pseudomonas aeruginosa* ATCC 10,145) and four Gram-positive bacteria *Staphylococcus aureus* 43,300, *Listeria monocytogenes* ATCC 25,152, and *Enterococcus faecalis* ATCC 43,845 were screened for sensitivity to inactivation with SBPTE. The bacterial strains were cultivated and incubated aerobically in nutrient broth overnight (NB) at 37 °C. Bacterial suspensions acquired for the assays were provided by diluting the cell biomass in 0.85% NaCl solution, and then, the cell number was adapted to 10^5^ cells/mL by dilution with saline. A stock suspension of SBPTE was prepared by suspending 10 mg/1 mL DEMSO.

The antibacterial competency of SBPTE was investigated in two ways: qualitative using the zone of inhibition assay (ZOI) and quantitative using the estimation of minimal inhibitory concentrations (MICs). For the ZOI assay, 100 µL of bacterial cell suspensions of the specific waterborne pathogen strains was uniformly dispersed onto the upper surface of Mueller–Hinton agar (MHA) plates, followed by the placement of impregnated sterilized discs with 50 µL of examined SBPTE onto the inoculated surfaces of MHA plates. The ZOI widths were accurately measured after 18–24 h of incubation at 37 °C. The antibacterial potential of SBPTE was compared to those of standard antibiotic discs (ciprofloxacin 30 μg) as positive controls and sterilized aqueous impregnated discs as negative controls (Radwan et al. 2020).

In microtiter plates, the bactericidal action of SBPTE was explored using the micro-dilution technique to evaluate the minimum inhibitory concentration (MIC). Each well (2.5 × 10^4^ CFU/well) acquired a suitable volume (100 µL) of customized bacterial culture, with the first well serving as a negative control. Then, apply a series of different stock solution concentrations of SBPTE ranging from 25 to 200 µg/mL in the other wells. The injected microtiter plate was wrapped with parafilm and incubated at 37 °C for 24 h for bacterial growth. After 24 h of incubation, the MIC of each bacterial suspension was established by pouring 20 µL of 0.2 mg/mL 2,3,5-triphenyltetrazolium chloride (TTC) marker dye into the microtiter plate wells. The injected microtiter plate was placed in an incubator at 37 °C for 30 min to notice any color changes. Tetrazolium salt is reduced to brilliantly pinkish-red formazan by the dehydrogenase of living bacteria (Silver 2011).

### Decontamination of some waterborne pathogen in artificially synthetic tap water

A proper concentration (log 6.0 CFU/mL) of bacterial suspension of the pathogenic strains designated above was transferred into a conical flask having 200 mL of sterile tap water to establish artificially contaminated water; the prepared SBPTE was employed as a disinfecting agent at its effective dose (150 mg/L). Before adding the proper concentration, the bacterial densities of each particular studied species were counted in synthetized polluted water samples using a spread plating count assay using stipulated chromogenic media for each bacterial (Hemdan et al. 2019). The container holding 200 mL of contaminated water carrying the effective dose of SBPTE was placed in the shaker at 150 rpm. A suitable volume (100 µL) of the flask was installed on top of the specified agar medium, and the specific type of pathogenic bacteria investigated was enumerated through different periods (0–90 min) (Cadillo-Benalcazar et al. 2020). Furthermore, a kinetic investigation using first-order equation was conducted to determine the relationship between time and dose of compound.

### Statistical study

All the experimental trials were conducted in triplicate and the collected data were represented as the mean ± standard deviation (*SD*). Error bars reveal *SD* of triplicate trials.

## Results and discussions

**Scheme 1:**
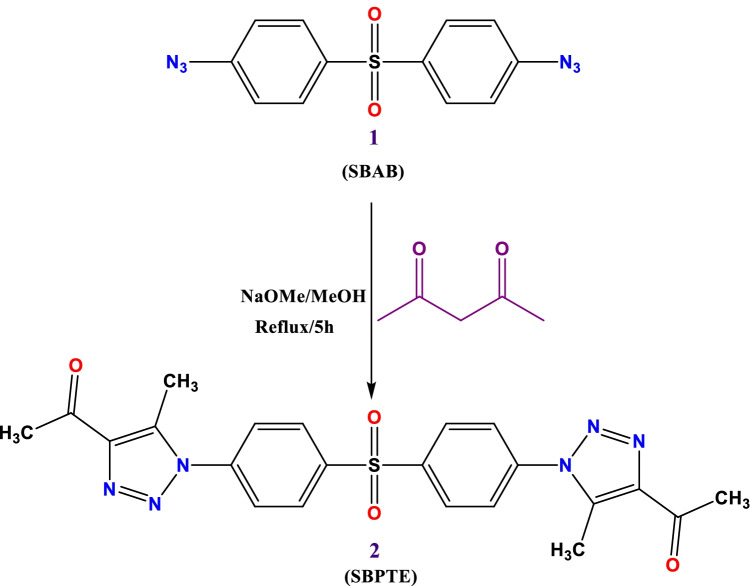
Synthesis of 1,1′-((sulfonylbis(4,1-phenylene))bis(5-methyl-1H-1,2,3-triazole-1,4-diyl))bis(ethan-1-one) (SBPTE)

**Fig. 1:**
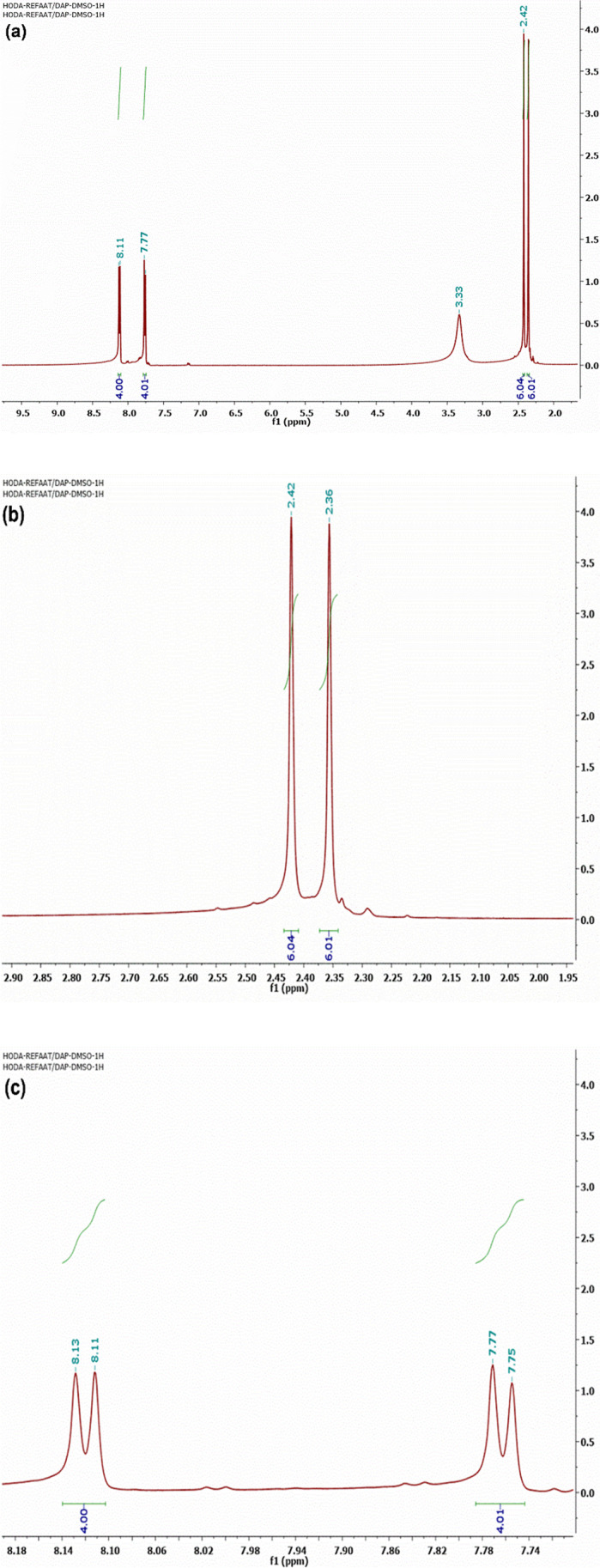
H.^1^NMR spectrum of SBPTE: **a** full spectrum and **b**, **c** magnified spectrum

**Fig. 2:**
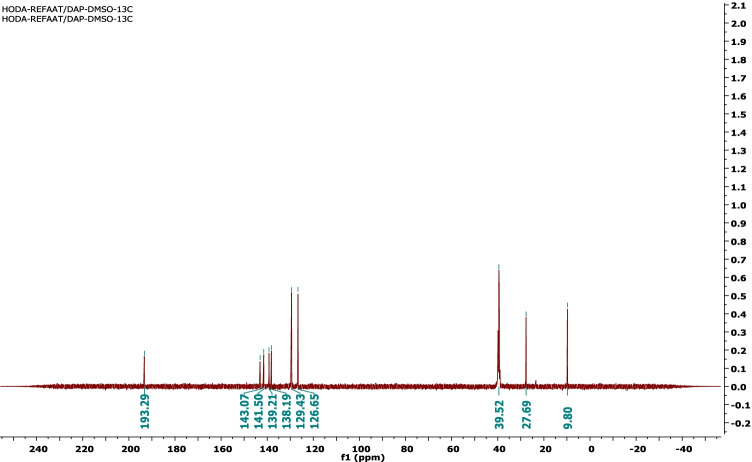
C.^13^NMR spectrum of SBPTE

The 4,4′-sulfonylbis(azidobenzene) (SBAB) was reacted with acetyl acetone in absolute methanol in the presence of sodium methoxide to get the desired compound 1,1′-((sulfonylbis(4,1-phenylene))bis(5-methyl-1*H*-1,2,3-triazole-1,4-diyl))bis(ethan-1-one) (SBPTE). Scheme 1 shows the synthesis procedure. Chemical structure of SBPTE was inferred from its spectral analysis. The H^1^NMR spectrum displayed at Fig. 1 shows two singlet signals at *δ* 2.36 and 2.42 for the protons of the four methyl groups and two doublet signals at *δ* 7.75 and 8.11 for the aromatic protons. C^13^NMR showed characteristic signals at *δ* 9.80 (CH_3_), 27.69 (CH_3_), 126.65, 129.43, 138.19, 139.21, 141.50, 143.07, and 93.29 (Ar–H) (Fig. 2). Thus, both H^1^NMR and C^13^NMR results elucidate the successful preparation of SBPTE.

### Adsorption performance

#### Effects of solution initial pH

**Fig. 3:**
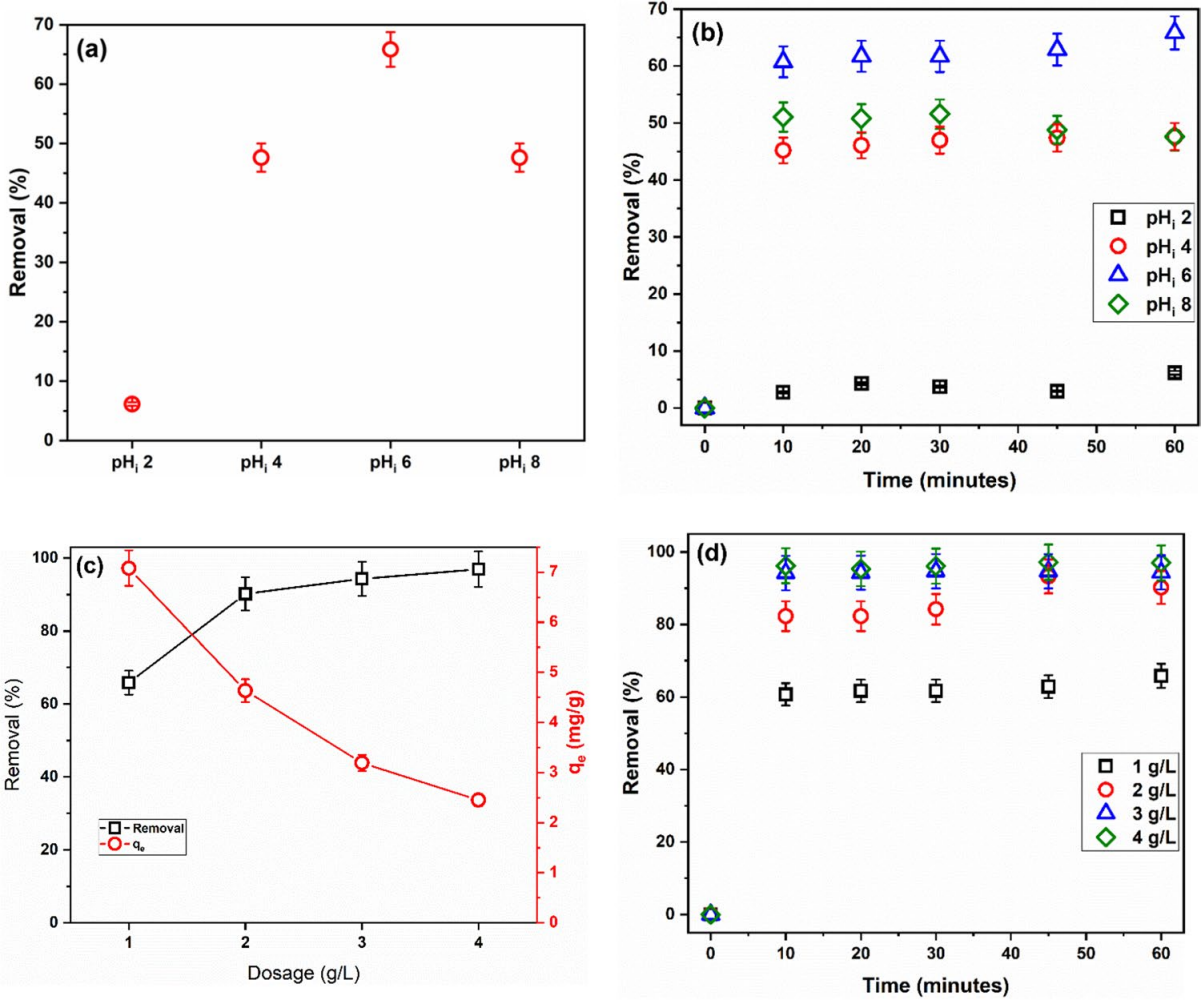
Removal of MG by SBPTE at different pH_*i*_ (**a**) after 1 h and (**b**) as a function of contact time (*C*_*i*_ = 10 mg/L, adsorbent amount 1 g/L), (**c**) variation of *q*_*e*_ and *R*% with SBPTE amount, and (**d**) ﻿removal of MG by different amounts of SBPTE as a function of contact time (*C*_*i*_ = 10 mg/L, adsorbent amount 1 g/L)

The initial pH (pH_*i*_) of the adsorptive solution is a key parameter that governs the extent of adsorption in adsorption systems that is dominated by electrostatic interactions. The pH_*i*_ controls the dissociation/undissociation of the functional groups of both adsorbent and adsorptive and, thus, causes repulsive or attractive interactions between the adsorbent and adsorptive. Therefore, investigating the effect of pH_*i*_ of adsorptive solution is paramount to optimize the adsorption process (Aljerf 2018). Figure 3 displays the effects of pH_*i*_ on the *R*% of MG.

Figure 3a shows clearly that pH_*i*_ affects significantly the adsorption of MG onto SBPTE. Specifically, little adsorption, only 6%, of MG was adsorbed at pH_*i*_ 2. A considerable increase in MG uptake by SBPTE was achieved by increasing the pH_*i*_ to 4 (*R*% = 48%). Increasing the pH_*i*_ to 6 brought about more enhancement in the adsorption efficiency (*R*% = 66%). But, further increase in the pH_*i*_ to 8 has disadvantageous effect on the adsorption process; the *R*% reduced to 48%. To be able to explain these results, the point of zero charge of SBPTE was determined by the salt addition method (Appel et al. 2003). The results displayed in Figure S2 indicate that SBPTE has a pH_PZC_ of 2.3. Thus, at pH_*i*_ less than 2.3, the surface of SBPTE is positively charged, and at pH_*i*_ greater than 2.3, the surface of SBPTE is negatively charged. On the other hand, MG is a cationic dye that has a pKa of 10.3, i.e., at pH < 10.3; MG bears positive charge in solution, while at pH > 10.3, MG exists in the neutral form (Igwegbe et al. 2021; Rubio-Clemente et al. 2021). Thus, at pH_*i*_ 2, the repulsion between the positively charged surface of SBPTE and the cationic MG results in the observed low *R*%. While at pH_*i*_ 4 and 6, the electrostatic interactions between the negatively charged surface of SBPTE and the cationic MG cause the observed increase in the *R*%. Notably, the *R*% is higher at pH_*i*_ 6 than at pH_*i*_ 4. This can be attributed to the competition between the H_3_O^+^ and the cationic MG for the adsorption sites on the surface of SBPTE. The concentration of H_3_O^+^ is higher at pH_*i*_ 4; consequently, the competition is higher and the removal is lower than at at pH_*i*_ 6. At pH_*i*_ 8, the hydroxide ions in the solution vie the electron-rich surface functional groups of SBPTE for binding to the cationic MG which results in decreasing the *R*%. Similar behavior was reported by Sartape et al. (2017) who used wood apple shell as a biosorbent for MG. Lin et al. (2021) used hexabromocyclododecane-polystyrene composites as adsorbent for MG and got similar behavior as well. Rubio-Clemente et al. (2021) reported similar effects of pH on MG adsorption by biochar obtained from *Pinus patula*.

Figure 3b shows also the effects of contact time on MG uptake by SBPTE at different pH_*i*_. Generally, it can be observed that the uptake takes place in two steps, a sharp uptake in the first 10 min followed by insignificant increase thereafter. Similar two-step adsorption process has been reported many times before (Alene et al. 2021; Güler et al. 2021; Igwegbe et al. 2021; Rubio-Clemente et al. 2021; Sartape et al. 2017). The high uptake in the first 10 min can be attributed to the presence of vast number of free and readily accessible adsorption sites on the surface of SBPTE and the good mass transfer induced by the high concentration of MG dye in the beginning of the adsorption process. As the contact time lapse, the number of free adsorption sites decreases and the mass transfer slows down as a result of the occupation of adsorption sites and the decrease of the MG dye concentration. Since the increase in MG dye uptake after 10 min was insignificant, it was considered as the equilibration time in this study.

#### Effects of amount of SBPTE

It is known that the amount of adsorbent is one of the parameters that govern the extent of adsorption; therefore, it should be studied to find the best dosage. The change of *R*% of MG with adsorbent dosage is displayed in Fig. 3c. A considerable increase in the removal percentage (from 65 to 90%) was achieved by increasing the dosage of SBPTE from 1 to 2 g/L. Such trend is readily understood as both the surface area and adsorption sites increase with increasing the adsorbent amount. Beyond 2 g/L, the enhancement in *R*% was minor. Numerically, 3 and 4 g/L of SBPTE remove 94 and 97% of MG dye, respectively. There are two probable reasons for this observation. First, high dosage of SBPTE might induce particles conglomeration which increases the diffusion path length and hides some adsorption sites. Second, since 2 g/L of SBPTE approaces full removal of MG dye, further increase in the dosage will not bring significant enhancement in the removal. These findings agree with earlier reports (Abu Elella et al. 2021; Alene et al. 2021; Güler et al. 2021; Igwegbe et al. 2021; Radwan et al. 2017; Sartape et al. 2017).

Figure 3c depicts that the amount of MG adsorbed per gram of SBPTE pursues contrariwise behavior to the *R*%. Increasing the amount of SBPTE decreases the *q*_*e*_ value. At low dosages of SBPTE, all adsorption sites are well unitized and occupied by MG molecules. As illustrated above, increasing the amount of SBPTE results in increasing the number of available adsorption sites; meanwhile, the concentration of MG dye is fixed, and consequently, some adsorption sites become unsaturated which caused the observed decrease in *q*_*e*_ value. These results agree with previous reports (Güler et al. 2021; Radwan et al. 2021). The time profile for the *R*% at different amounts of SBPTE (Fig. 3d) resembles that of the different pH_*i*_; the adsorption process was very fast in the first 10 min and then remained almost constant.

#### Adsorption kinetics modeling

**Fig. 4:**
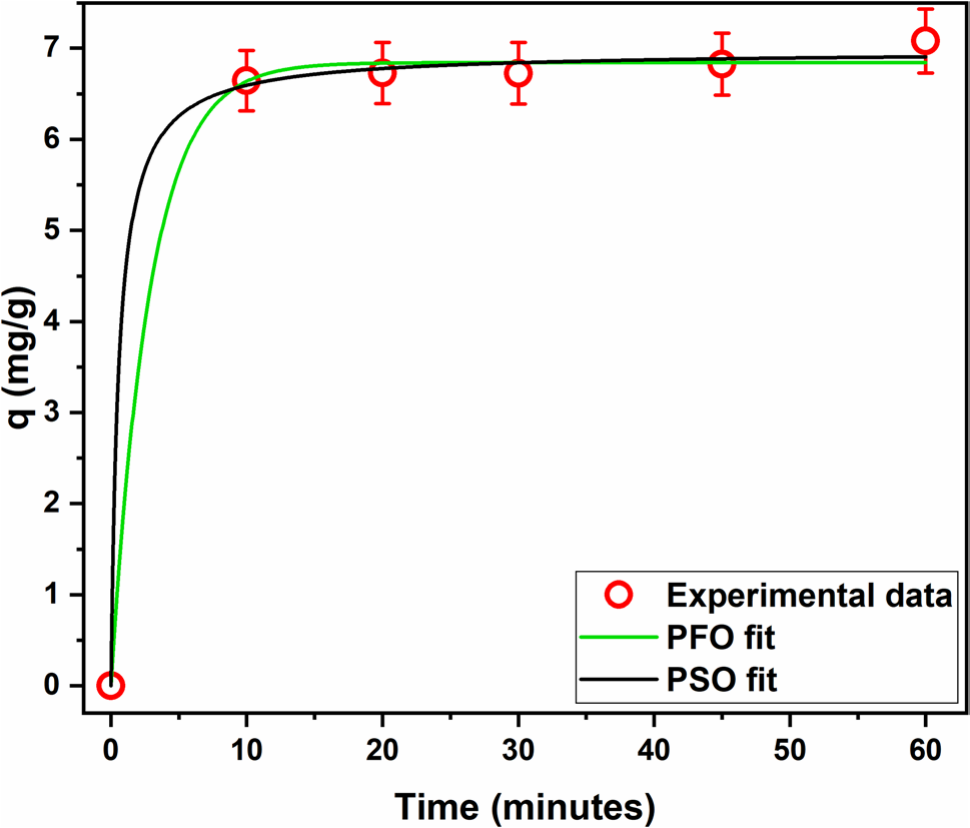
Experimental adsorption kinetics of MG onto SBPTE and fitted kinetic models. *C*_*i*_ = 10 mg/L, adsorbent amount 1 g/L, pH_*i*_ = 6

Modeling the adsorption kinetics gives useful information about the rate and the underlying mechanism of the adsorption process. For this purpose, the nonlinear forms of PFO and PSO equations were applied. Fitting of the experimental kinetic data to the studied models is given in Fig. 4, and the derived kinetic parameters are listed in Table 1.

**Table 1:** Calculated kinetic models’ parameters

PFO		PSO	
***q***_***e*,exp**_		7.08	
***R***^**2**^	0.998	***R***^**2**^	0.999
***χ***^**2**^	0.021	***χ***^**2**^	0.013
**RMSE**	0.144	**RMSE**	0.114
**k**_**1**_	0.35 ± 0.08	**k**_**2**_	0.25 ± 0.12
***q***_***e***_	6.84 ± 0.07	***q***_***e***_	6.97 ± 0.10

Analysis of the data shown in Table 1 indicates that both PFO and PSO can describe the kinetic data. However, the PSO model has an *R*^2^ value closer to 1 and error functions closer to zero than the PFO model. Also, the value of *q*_*e*_ calculated by the PSO is closer to the experimental value than the PFO model. Thus, the PSO is more appropriate than the PFO for describing the kinetic data. Therefore, it is likely that the adsorption process involves physicochemical interactions, exchange, or sharing of electrons between the functional groups of SBPTE and the MG molecules (chemisorption) and that this step is the rate-limiting step. Similar findings have been reported earlier for the adsorption of MG on other adsorbents (Güler et al. 2021; Igwegbe et al. 2021; Tohidi et al. 2021).

**Fig. 5:**
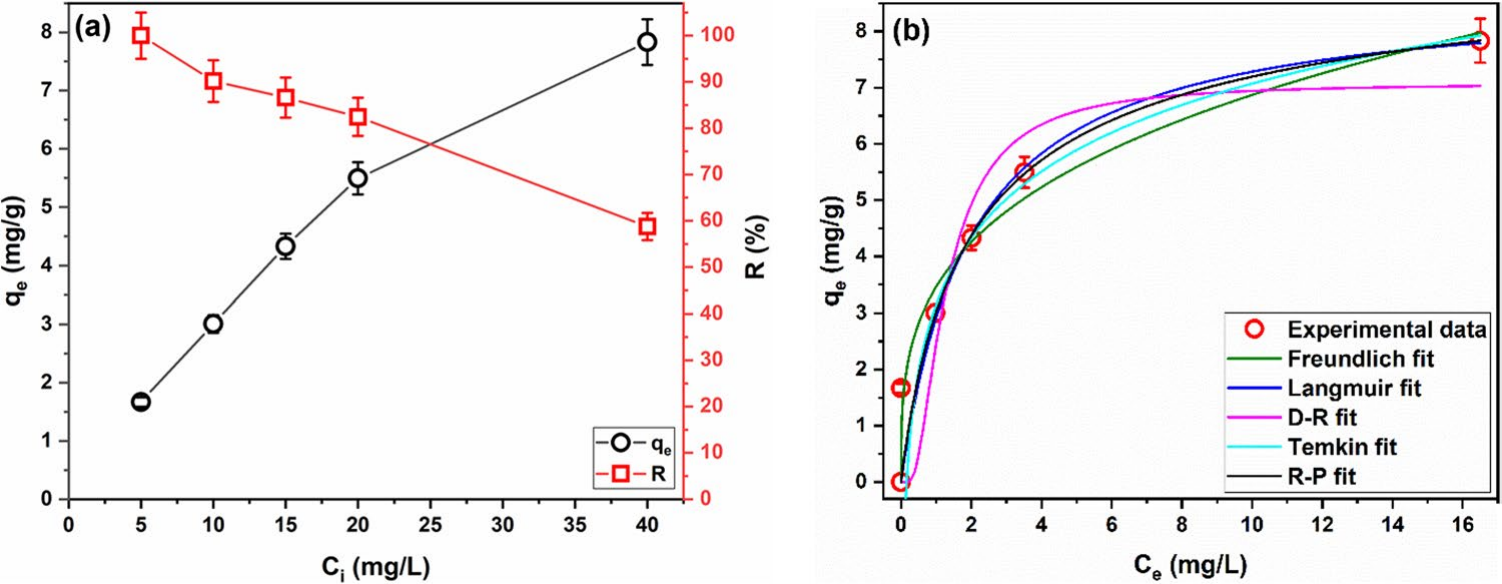
**a** Variation of *q*_*e*_ and *R*% with initial concentration of MG dye, and **b** experimental adsorption isotherm and fitted isotherm models. Contact time 1 h, adsorbent amount 3 g/L, pH_*i*_ = 6

The effect of the *C*_*o*_ of MG dye on the *R*% and the amount adsorbed per unit mass of SBPTE were evaluated and are displayed in Fig. 5a. Increasing the initial concentration of MG causes an incessant decrease in the *R*% and an incessant increase in *q*_*e*_. Similar findings have been reported before (El Bendary et al. 2021; Güler et al. 2021; Sartape et al. 2017; Tohidi et al. 2021). At low *C*_*i*_ of MG (5 mg/L), the number of adsorption sites will be sufficient to achieve complete removal of the dye and there will be a number of unsaturated sites resulting in low *q*_*e*_. As *C*_*i*_ of MG increased, the mass transfer resistance between aqueous and solid phase decreases which leads to better utilization of the adsorption sites and subsequently increasing the *q*_*e*_ value. Meanwhile, the saturation of adsorption sites results in high percentage of free MG in the solution which causes the decline of the *R*%.

With the sake of understanding the adsorption mechanism, surface properties, and affinities of SBPTE, determining the adsorption capacity of SBPTE, and finding the best model that describes the equilibrium adsorption data, the adsorption isotherm was experimented and the obtained data was analyzed using different models. Figure 5b exhibits the experimental adsorption isotherm and the fitting plot to the different isotherm models tested in this work. The values of driven models’ parameters and their error functions are given in Table 2; according to the values of *R*^2^, the R–P, Langmuir, and Temkin give similar fit to the experimental adsorption isotherm and are better than Freundlich and D–R models. However, the values of error functions were lowest for Langmuir revealing that it is the most appropriate model for describing the experimental adsorption isotherm. Langmuir model postulates that the surface of adsorbent contains a finite number of energetically homogeneous adsorption sites, only one layer of adsorbate covers the surface of the adsorbent, and the adsorbate does not interact with each other’s. Therefore, the adsorption of MG dye on SBPTE fulfils these assumptions. In other words, the surface of SBPTE has a finite number of energetically equal adsorption sites and MG forms a monolayer coverage on the surface of SBPTE. The values of separation factor (*R*_L_) were calculated by Eq. 3 to analyze the essential characteristics of Langmuir isotherm and are given in Table 2.

**Table 2:** Calculated isotherm models’ parameters

Freundlich		Langmuir		D‒R		Temkin		R‒P	
***R***^**2**^	0.918	***R***^**2**^	0.928	***R***^**2**^	0.881	***R***^**2**^	0.927	***R***^**2**^	0.929
***χ***^**2**^	0.803	***χ***^**2**^	0.701	***χ***^**2**^	1.158	***χ***^**2**^	0.711	***χ***^**2**^	0.927
**RSME**	0.896	**RSME**	0.837	**RSME**	1.076	**RSME**	0.843	**RSME**	0.963
***K***_**F**_	3.46 ± 0.58	***K***_**L**_	0.50 ± 0.21	***β***	0.36 ± 0.15	***b***_**T**_	1458.43 ± 349.15	***K***_**R–P**_	5.17 ± 6.58
***n***	3.36 ± 0.89	***q***_**m**_	8.74 ± 1.19	***q***_**D–R**_	7.08 ± 0.87	***A***_**T**_	6.32 ± 4.83	***a***	0.72 ± 1.76
		***R***_**L**_	0.29–0.05					***g***	0.94 ± 0.40

3 $${\text{R}}_\text{L}\text{=}\frac{1}{{1+K_\text{L}}{\text{C}_\text{e}}}$$

The adsorption is favorable when the value of *R*_L_ ranges between zero and 1, unfavorable if *R*_L_ is larger than 1, linear if *R*_L_ equals 1, and irreversible if *R*_L_ equals zero. In this study, the calculated values of R_L_ (0.29–0.05) indicate that the adsorption of MG on SBPTE is a favorable process.

#### Probable adsorption mechanism

The adsorption of cationic dyes can occur via pore filling, hydrogen bonding formation, anionic and cationic exchange, electrostatic interactions, n–π interaction, and π–π interaction (Tran et al. 2017). SBPTE is a non-porous material (see Fig. 6a and b), and the structures of both SBPTE (Scheme 1) and MG dye (Figure S1) do not contain exchangeable anions or cations or hydrogen atoms that can undergo hydrogen bonding formation. Therefore, pore filling, hydrogen bonding formation, and the anionic and cationic exchange can be ruled out.

**﻿Fig. 6:**
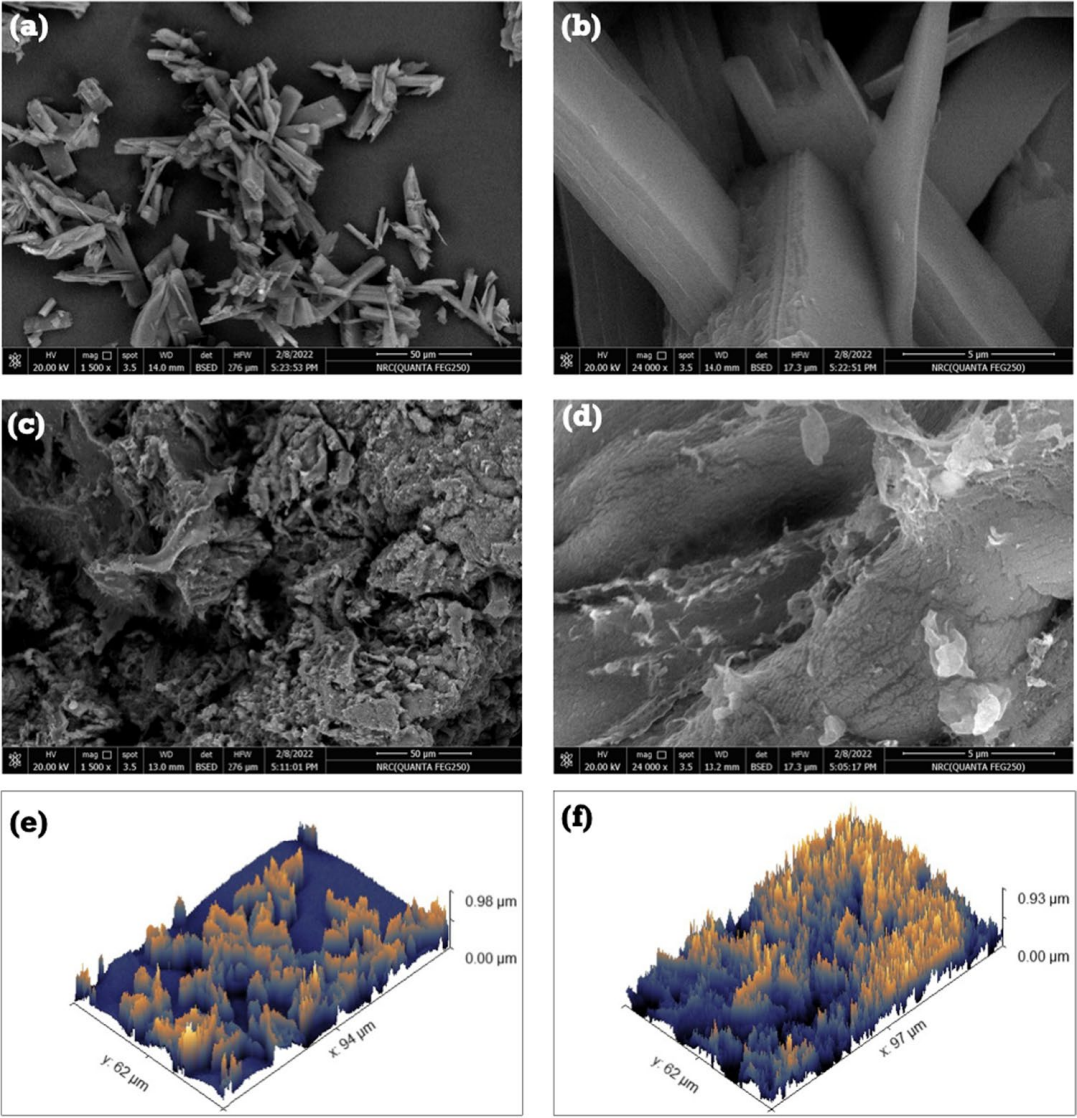
SEM of SBPTE at low and high magnifications **a**, **b** before and **c**, **d** after MG dye adsorption. And roughness structure of SBPTE **e** before and **f** after MG dye removal

The role of electrostatic interactions has been discussed in details in the section “Effects of solution initial pH” which manifested the important role of electrostatic interactions in the adsorption process. Another type of probable interaction between SBPTE and MG dye is the n–π interactions. SBPTE has several oxygen and nitrogen atoms that have lone pair of electrons. These oxygen and nitrogen atoms can act as electron donors while the aromatic rings of MG act as electron acceptors (Rani et al. 2021). Finally, both SBPTE and MG contain several aromatic rings which are electron-rich zones that could bring a π–π interactions between the π-electrons in SBPTE and the π-electrons in the aromatic rings of MG resulting in a stacking effect of MG onto SBPTE. Therefore, both n–π interactions and π–π interactions could contribute to the adsorption of MG on SBPTE. Overall, the adsorption mechanism could be a combination of electrostatic, n–π, and π–π interactions. Scheme 2 summarizes the probable adsorption mechanism of MG on SBPTE.

**﻿Scheme 2:**
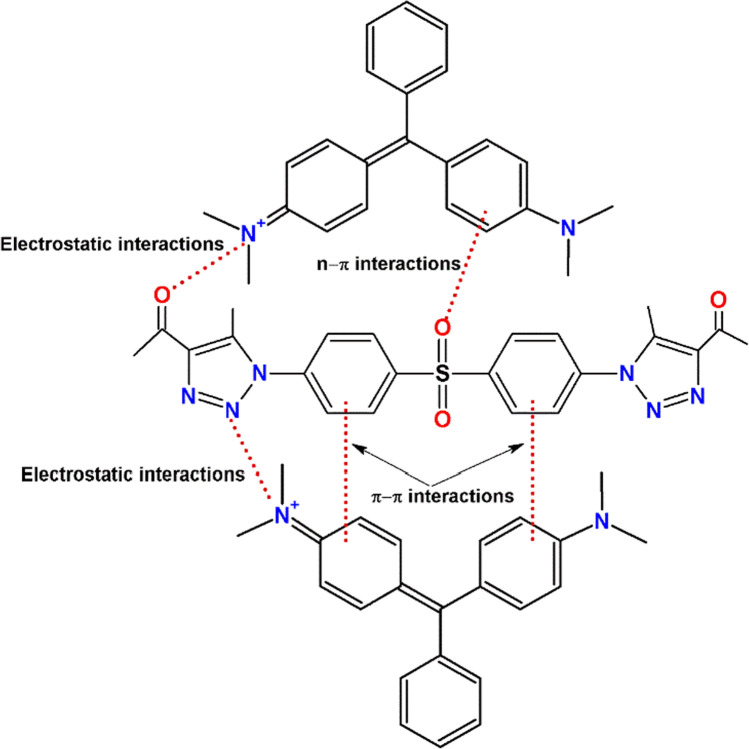
Probable mechanism for the adsorption of MG dye on SBPTE

The surface structure of the as-prepared SBPTE before and after MG removal was examined. Figure 6 displays the SEM images at low and high magnifications. The as-prepared SBPTE powder sample (Fig. 6a and b) consists of rather uniform and smooth surface rod-like micro-structures with a length of 1.2 to 2.5 µm. After MG dye adsorption, it can be clearly seen that SBPTE exhibits morphological structure with no clear edges due to the roughly deposition of dye onto the surface of SBPTE. The presence of MG dye in a liquid solution facilitates the disintegration of the grains and agglomerates of SBPTE into bulk particles with no clear edges (Fig. 6c and d). In addition, it is remarkable that the surface of SBPTE changed from smooth (before MG adsorption) to rough (after MG adsorption) likely owing to the huge deposition of many molecules of MG dye. To further confirm this observation, the roughness of SBPTE before and after MG dye adsorption was assessed. The roughness behavior of SBPTE is shown in Fig. 6e and f before and after MG adsorption, respectively, along with the values of the roughness parameters. From Table 3, it can be noticed that, after MG dye adsorption, the average roughness (*R*_a_) increased from 51.9 to 61.3 nm, the maximum height of the roughness (*R*_t_) increased from 69.7 to 82.1 nm, and the maximum roughness valley depth (*R*_v_) changes from 221.7 to 251.4 nm. Moreover, it could be noticed that the value of average maximum height of the roughness (*R*_tm_) is much higher than the average maximum roughness valley depth (*R*_vm_) for each composition. This behavior refers to the component of the surface topography. In other words, the surface morphology is composed of notches and heights. Both components act to facilitate interacting with the MG dye. While the heights might prefer to hock the MG dye molecules, the notches could facilitate their trapping. Thus, the high values of heights might encourage interacting with MG dye molecules. Moreover, the increasing of topographical signs for the composition after treatment might indicate the high adsorption of dye molecules within the waviness of the composition’s surface (Xing et al. 2019).

**Table 3:** Surface roughness parameters of SBPTE

	***R***_**a**_	***R***_**q**_	***R***_**t**_	***R***_**v**_	***R***_**p**_	***R***_**tm**_	***R***_**vm**_	***R***_**pm**_
Before adsorption	51.9	69.7	477.5	221.7	255.7	345.0	160.3	184.7
After adsorption	61.3	82.1	511.1	251.4	259.7	419.9	204.3	215.6

*R*_*a*_ average roughness, *R*_*q*_ root mean square roughness, *R*_*t*_ maximum height roughness, *R*_*v*_ maximum roughness valley depth, *R*_*p*_ maximum roughness peak height, *R*_*tm*_ average maximum height of roughness

### Antibacterial evaluation

Quantitative and qualitative antibacterial investigations were practiced to evaluate the antimicrobial activities of the prepared SBPTE towards six pathogenic strains (*E. coli*, *E. enterica*, *P. aeruginosa*, *S. aureus*, *L. monocytogenes*, and *E. faecalis*). The results of biological activities are tabulated in Table 4. The control modes (SDW) did not own bactericidal activity against Gram-positive bacteria, while the prepared SBPTE prevented the growth of all tested pathogens, but with differing degrees. Furthermore, the prepared SBPTE was significantly more effective against Gram-negative bacteria than Gram-positive bacteria, with ZOI diameters of 25 ± 1.8, 23 ± 2.1, 27 ± 1.3, 20 ± 2.2, 17 ± 2.4, and 21 ± 1.5 mm for *E. coli*, *E. enterica*, *P. aeruginosa*, *S. aureus*, *L. monocytogenes*, and *E. faecalis*, respectively. The diameters of ZOI of the prepared SBPTE for all tested bacterial species were larger than the ZOI created by the positive controls (ciprofloxacin) in the antibacterial testing. According to these observations, the prepared sulfone biscompound had significantly superior antimicrobial properties than ciprofloxacin against all harmful microorganisms tested (El Nahrawy et al. 2021).

**Table 4:** Antibacterial potentialities (mean ± *SD*) of SBPTE against some waterborne pathogens

	SBPTE		Ciprofloxacin
	ZOI (mm)	MIC (µg/mL)	ZOI (mm)
*E. coli* O175:H7	25 ± 1.8	125 ± 21	12 ± 2.0
*S. enterica*	23 ± 2.1	125 ± 16	11 ± 1.8
*P. aeruginosa*	27 ± 1.3	100 ± 11	11 ± 2.1
*S. aureus*	20 ± 2.2	150 ± 26	9 ± 1.4
*L. monocytogenes*	17 ± 2.4	175 ± 9	10 ± 1.6
*E. faecalis*	21 ± 1.5	150 ± 18	10 ± 2.2

Moreover, the MIC values of the prepared SBPTE were 125, 125, 100, 150, 175, and 150 µg/mL, respectively, for *E. coli*, *E. enterica*, *P. aeruginosa*, *S. aureus*, *L. monocytogenes*, and *E. faecalis* (Table 4). These results uncovered that SBPTE has significant antimicrobial activity. In some cases, the activity of SBPTE was higher in Gram-negative bacteria than in Gram-positive bacteria. Lv et al. (2014) revealed that 1,2,3-triazole-derived naphthalimides had energetic biocidal effect against microbial species, as antimicrobial methods exhibited that such compounds indicated better antibacterial action against *E. coli* than used reference drugs (norfloxacin and chloromycin).

### Structure activity relationships

Sulfone compounds, also referred as cyclic sulfones, are a class of organosulfur compounds that are particularly interested to chemists. Certain classes of sulfone-containing molecules and their analogues have numerous uses in various sectors, such as biomedicine, but they also involve a variety of biological functions. Sulfones and their derivatives are employed as pharmacological and polymeric agents in a variety of industries. Different disorders such as dermatitis herpetiformis, leprosy, yellow fever, and others are addressed with sulfone-containing drugs. Because of its varied significant therapeutic activities such as biological, antimalarial, antimicrobial, antitumor, anti-HIV, and anti-inflammatory, investigators have been working very hard into manufacturing numerous multiple kinds of sulfone molecules (Alam et al. 2018). Furthermore, previously published academic articles and journals have confirmed that the 1,2,3-triazole bisulfone compounds has significant antimicrobial properties, as it can destroy the whole genome content in microbial species containing antibiotic resistance genes, resulting in disabling these genes at the end (El Malah et al. 2022). According to previous research, the compound can efficiently block DNA replication and thus has antimicrobial effects (Lv et al. 2014). Targeting mechanisms that induce evolved antibiotic resistance is a potential substitute. The SOS response, which is a bacterial DNA damage response route, is one of them. Many antibiotics trigger the SOS response, either immediately (e.g., fluoroquinolones) or indirectly (e.g., antibiotics that target key cellular and metabolic activities) (Mo et al. 2016). The SOS response is highly maintained between microbial pathogens and includes a large number of genes (about 40 in E. coli, for instance). Translesion DNA polymerases facilitate mutagenesis, restriction enzymes mobilize antibiotic resistance genes, and proteins that mediate persistence, biofilm formation, or promote and market antibiotic evasion are all examples of these proteins (Gotoh et al. 2010).

### Influence of the effective dose of SBPTE and exposure time in the disinfection of some waterborne pathogens

**Fig. 7:**
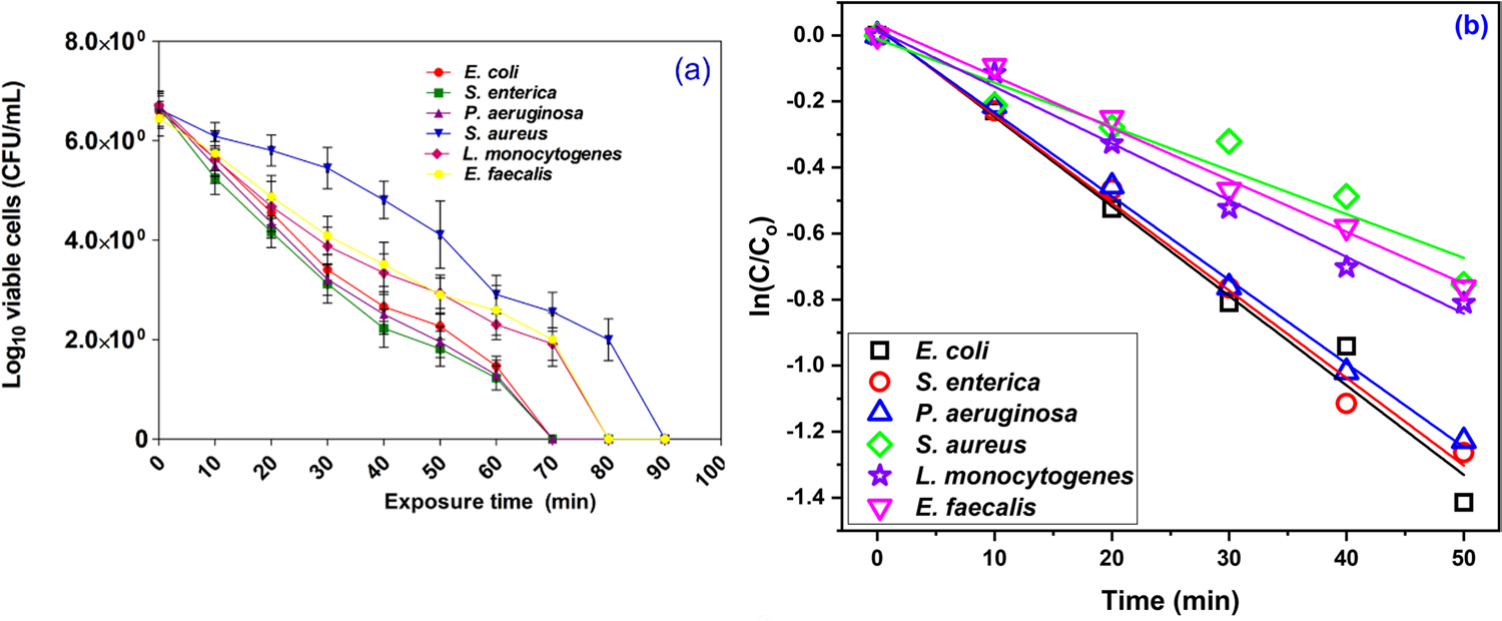
**a** Decontamination efficiency and **b** kinetic of the inactivation of some waterborne pathogenic bacterial species using 150 mg/L of SBPTE

To further clarify the role of SBPTE in the decontamination of some pathogenic bacteria, disinfection trials with the addition of different contact times into the reaction system (to inactivate the corresponding waterborne pathogenic species) were conducted. As shown in Fig. 7, a significant inactivation rate was witnessed for all tested bacteria at which all examined bacterial strains were completely eradicated using 150 mg/L at varying exposure times depending on the nature and type of bacterial species and the resistance of each bacterium to SBPTE. Furthermore, the experimental results revealed that the required time for deactivating Gram-negative species, including *E. coli*, *E. enterica*, and *P. aeruginosa*, was 70 min, where log 6 counts of cell densities were absolutely removed. On the other hand, log 6 of Gram-positive species such as *S. aureus*, *L. monocytogenes*, and *E. faecalis* was diminished after 90, 80, and 80 min, respectively. These results explicate that Gram-negative bacterium can be easily and fastly eliminated than Gram-positive ones. This is attributed to the structure of the Gram-negative cell wall, which consists of a thin layer of peptidoglycan (El Nahrawy et al. 2019; Sun et al. 2021). The interaction between SBPTE and the cell wall promotes its destruction, making the outflow of intracellular substances, leading to the death of bacteria (El Nahrawy et al. 2019; Sun et al. 2021).

The decay rate of some waterborne pathogenic bacterial species using the effective dose of SBPTE is displyed in Fig. 7b and Table S1. The results depicted that the decay rate of Gram-negative bacteria (*E. coli*, *E. enterica*, and *P. aeruginosa*) was swift. While when Gram-positive species (*S. aureus*, *L. minocytogenes*, and *B. subtilis*) were involved, the decay rate was slow. These results are compatible with the results of the disinfection process, which confirmed that Gram-negative bacteria required less time to be completely eradicated than Gram-positive bacteria.

## Conclusions

Developing a versatile material that can remove dyes and eradicate pathogenic microorganism was targeted in this study. The 1,1′-((sulfonylbis(4,1-phenylene))bis(5-methyl-1H-1,2,3-triazole-1,4-diyl))bis(ethan-1-one) was successfully synthesized. Its chemical structure was verified based on spectral data. The performance of the synthesis of this new sulfone biscompound in the removal of cationic dye and disinfection of contaminated water was evaluated deeply. According to the data obtained from current research, the following can be concluded:

- The adsorption study disclosed the dependence of the amount of MG removed on the solution initial pH and amount of SBPTE.
- Additionally, it can be disclosed that the adsorption process was favorable, and monolayer, and that SBPTE has energetically equal adsorption sites.
- The adsorption of MG on SBPTE occurred, likely, via a combination of electrostatic, n–π, and π–π interactions.
- Results of the biological activity study displayed that SBPTE exhibited an energetic bactericidal effect. Besides, SBPTE could wholly deactivate all studied bacterial pathogens.

By and large, this study showed that SBPTE might be applied for water decontamination from toxic dyes and pathogenic microbes. Future work should consider the preparation of easily separable adsorbent that can remove different types of pollutants from aqueous medium simultaneously.

## Supplementary Information

Below is the link to the electronic supplementary material.

Supplementary file1 (PDF 267 KB)

## Data Availability

The datasets generated and analyzed during the current study are not publicly available but are available from the corresponding author on reasonable request.
