# Supplementary material for: A dual-functional sulfone biscompound containing 1,2,3-triazole moiety for decolorization and disinfection of contaminated water
Source: Environ Sci Pollut Res Int. 2022 Jun 8;29(51):77238–52. doi: 10.1007/s11356-022-20932-5 (PMC9581830; doi:10.1007/s11356-022-20932-5)
Supplement: Supplementary file 1 — Supplementary file1 (PDF 267 KB) [file 11356_2022_20932_MOESM1_ESM.pdf]

Supporting information

A dual-functional sulfone biscompound containing 1,2,3-triazole moiety for decolorization and disinfection of contaminated water

Emad K. Radwan [a*], Huda R. M. Rashdan [b], Bahaa A. Hemdan [a], Asmaa A. Koryam [a], and Mehrez E. El-Naggar [c*]

[a] Water Pollution Research Department, National Research Centre, 33 El Buhouth St, Dokki, 12622 Giza, Egypt.

[b] Chemistry of Natural and Microbial Products Department, Pharmaceutical and Drug Industries Research Institute, National Research Centre, 33 El Buhouth St, Dokki, 12622 Giza, Egypt.

[c] Institute of Textile Research and Technology, National Research Centre, 33 El Bohouth St, Dokki, Giza, 12622, Egypt.

Contents

Adsorption kinetics models. 2

Adsorption isotherm models. S2

Error functions. S2

Figure S1. Structure and some properties of MG dye. S4

Figure S2. pH_{PZC} of SBPTE. S5

Table S1. First order rate constants for the inactivation of some waterborne pathogenic bacterial by SBPTE. S6

* Corresponding authors. E-mail addresses: emadk80@gmail.com (Emad K. Radwan) and mehrez_chem@yahoo.com (Mehrez E. El-Naggar).

Adsorption kinetics models.

Pseudo-first-order $$q_t = q_e\,(1 - e^{-k_1 t})$$ (S1)

Pseudo-second-order $$q_t = \frac{k_2 q_e^2 t}{1 + k_2 q_e t}$$ (S2)

where k_1 (1/min) is the PFO rate constant, k_2 (g/mg.min) is the PSO rate constant, respectively, and t (min.) is the contact time.

Adsorption isotherm models.

Freundlich $$q_e = K_f\, C_e^{1/n}$$ (S3)

Langmuir $$q_e = \frac{q_L K_L C_e}{1 + K_L C_e}$$ (S4)

Dubinin–Radushkevich $$q_e = q_{D\text{-}R}\, e^{\left[-\beta\left(\mathrm{RTLn}\left(1+\frac{1}{C_e}\right)\right)\right]}$$ (S5)

Temkin $$q_e = \left(\frac{\mathrm{RT}}{b_T}\right) lnA_T C_e$$ (S6)

Redlich–Peterson $$q_e = \frac{K_{R\text{-}p}\, C_e}{1 + a_{R\text{-}P}\, C_e^{g}}$$ (S7)

where K_F (mg$^{(1-1/n)}$L$^{(1/n)}$/g) and n (–) are Freundlich constants; q_L (mg/g) is the Langmuir monolayer saturation capacity, and K_L (L/mg) is the Langmuir equilibrium constant; $q_{D–R}$ (mg/g) is the D–R maximum adsorption capacity, β (kmol^2/J^2) is a D–R constant, R is the ideal gas constant (kJ/mol.K), and T (K) is the thermodynamic temperature; b_T (kJ/mol) and A_T (L/g) are Temkin constants related to the adsorption heat, and equilibrium binding, respectively; $K_{R–P}$ (L/mg) and $a_{R–P}$ (L/mg) are R–P constants related to the adsorption capacity, and the affinity of the binding sites, respectively, and g (–) is an exponent related to the adsorption intensity.

Error functions.

Coefficient of determination $$R^2 = \frac{\sum \left(q_{e,cal} - \bar{q}_{e,exp}\right)^2}{\sum \left(q_{e,cal} - \bar{q}_{e,exp}\right)^2 - \sum \left(q_{e,cal} - q_{e,exp}\right)^2}$$ (S8)

Chi-square $$\chi^2 = \sum_{i=1}^{N} \left[\frac{\left(q_{e,exp} - q_{e,cal}\right)^2}{q_{e,cal}}\right]$$ (S9)

Root-mean-square error

$$RMSE = \sqrt{\frac{1}{N-M}\sum_{i=1}^{N}(q_{e,measured} - q_{e,model})^2} \quad \text{(S10)}$$

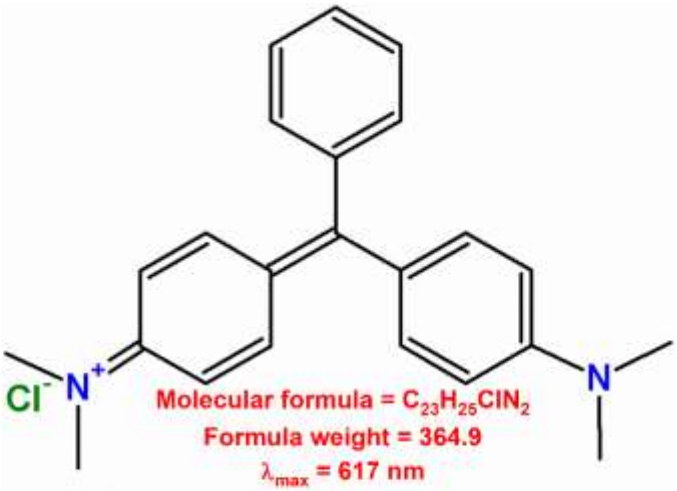

Figure S1. Structure and some properties of MG dye.

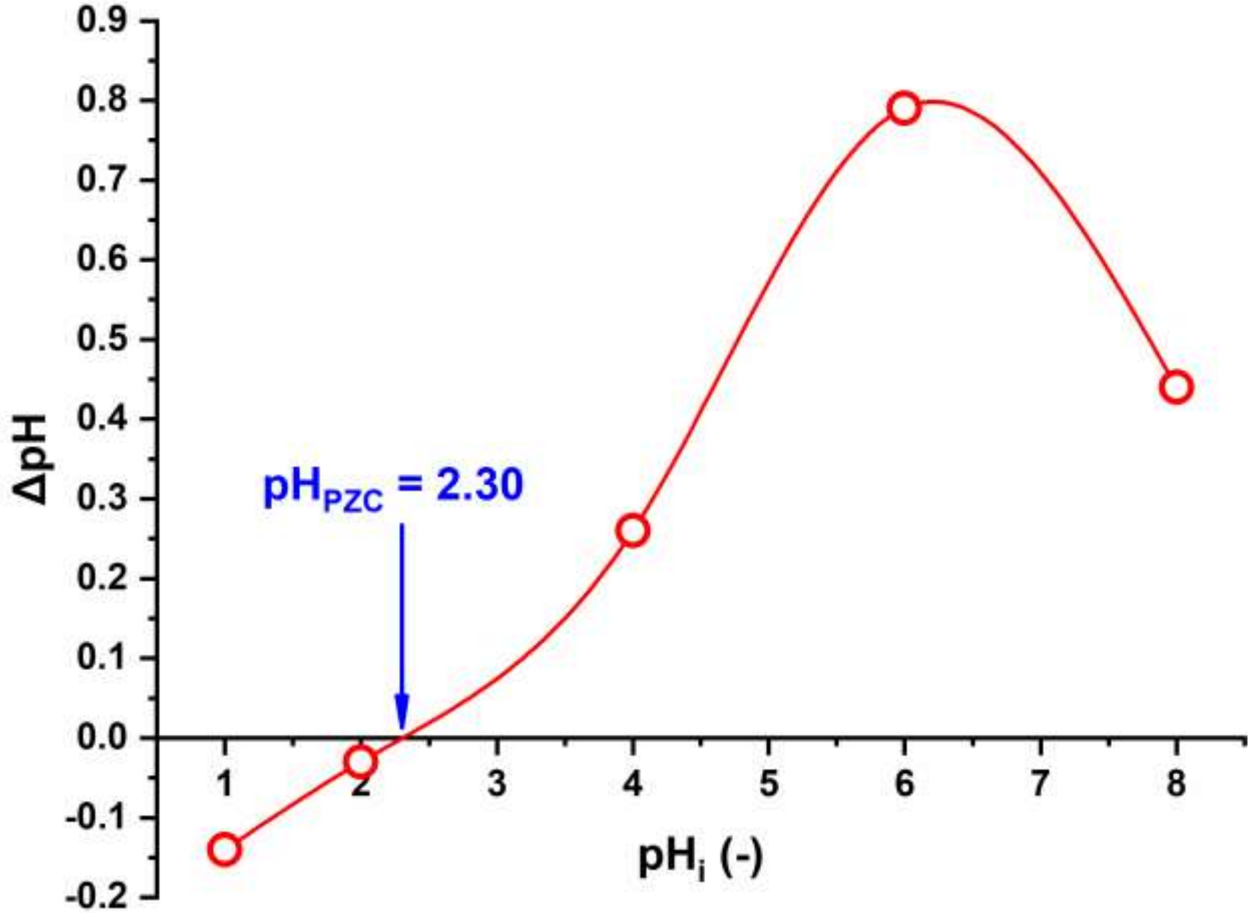

Figure S2. pH_{PZC} of SBPTE.

Table S1. First order rate constants for the inactivation of some waterborne pathogenic bacterial by SBPTE.

	**k (min^{-1})**	**R^2**
E. coli	-0.027	0.983
S. enterica	-0.026	0.992
P. aeruginosa	-0.025	0.997
S. aureus	-0.013	0.934
L. monocytogenes	-0.017	0.991
E. faecalis	-0.016	0.990
